# An XGBoost-Based Morphometric Classification System for Automatic Subspecies Identification of *Apis mellifera*

**DOI:** 10.3390/insects17010027

**Published:** 2025-12-24

**Authors:** Miaoran Zhang, Yali Du, Xiaoyin Deng, Jinming He, Haibin Jiang, Yuling Liu, Jingyu Hao, Peng Chen, Kai Xu, Qingsheng Niu

**Affiliations:** 1 Key Laboratory of Pathobiology, Ministry of Education, Jilin University, Changchun 132108, China; mrzhang20@mails.jlu.edu.cn; 2 Jilin Provincial Key Laboratory of Bee Genetics and Breeding, Jilin 132108, China; duyali2000@yeah.net; 3 Apiculture Science Institute of Jilin Province, Jilin 132108, China; 17836500979@163.com (X.D.); hejinmingmifeng@163.com (J.H.); jhb18047513706@163.com (H.J.); 15843267968@163.com (Y.L.); haojingyu2025@163.com (J.H.)

**Keywords:** *Apis mellifera*, machine learning, extreme gradient boosting (XGBoost), subspecies identification

## Abstract

The conservation and breeding of the western honey bee (*Apis mellifera*) is central dependent on accurate subspecies assignment, but the most commonly used methods are labor-intensive classical morphometrics and costly molecular assays. We developed an XGBoost-based classification framework using a compact set of routinely measurable characters. A curated dataset of labeled workers was measured under harmonized protocols; features were screened according to embedded importance, and model performance was assessed using five-fold cross-validation, outperforming standard machine learning baselines. The resulting model using only the top 10 characters—primarily forewing venation angles and abdominal plate metrics—achieved high performance (accuracy = 0.98; F1 = 0.99) and an area under the receiver operating characteristic curve (AUC) of 0.99 (95% CI = 0.995–0.999). SHAP analyses confirmed the discriminatory contributions of these features, while error inspection suggested that misclassifications were concentrated in morphologically overlapping lineages. The model’s performance supports its use as a rapid triage tool alongside genetic testing, providing a scalable and interpretable tool for researchers to create and deploy custom morphometric models, demonstrated here for *A. mellifera* but portable to other insect taxa.

## Simple Summary

The reliable identification of honey bee subspecies is important for their breeding and conservation, but common approaches can be slow or expensive. We measured a compact set of routine body traits—mainly forewing angles and abdominal plate sizes—in worker bees collected under a standard protocol. Using these measurements, we built a small, easy-to-use classification tool that assigns subspecies with very high accuracy. The tool also shows which traits drive each decision so that users can understand why a specimen was assigned to a group. It runs quickly on a regular computer, accepts local data, and produces clear plots and a short list of key traits. The same steps can be retrained on new regional datasets. Our results show that routine measurements, combined with an accessible computer-based approach, can support fast screening in the lab or field and help prioritize samples for follow-up genetic testing.

## 1. Introduction

The western honey bee (*Apis mellifera*) is among the most frequently observed floral visitors in natural habitats worldwide and supports pollination services essential to agriculture and ecosystem function [1], meaning that the accurate identification of *Apis mellifera* subspecies is fundamental to sustainable breeding, resource management, and conservation.

Classical subspecies diagnosis relies on morphometrics—most notably Ruttner’s framework that uses standardized measurements of forewing venation and other body traits [2]. This approach established a common taxonomic language and remains widely used. However, it is labor-intensive—requiring manual landmarking and expert curation—and is sensitive to environmental and developmental influences on morphology, which can complicate discrimination among closely related lineages [3,4,5]. In practice, consistent results depend on robust reference datasets and harmonized protocols across laboratories [2].

Additional challenges to conservation include the extensive human-mediated movement of bees and the species’ mating biology. Queens mate on the wing at drone congregation areas (DCAs), which aggregate drones from many colonies and can promote admixture when non-native stocks are present [6,7,8]. As a result, subspecies boundaries may become porous, with mixed ancestry reported in parts of Europe and elsewhere [9,10,11]. These factors make it difficult to maintain locally adapted populations without reliable, scalable identification tools.

Molecular methods provide high resolution but currently lack a globally standardized, broadly adopted diagnostic panel. Regional SNP assays and supervised machine-learning classifiers have shown high accuracy when assigning European subspecies and detecting introgression. However, training data are often geographically limited, and these approaches require genotyping infrastructure [9,12,13,14,15]. Mitochondrial DNA markers are informative for maternal lineages but do not capture nuclear introgression; therefore, alone, they are insufficient for ancestry assignment [16,17,18].

Recent advances in machine learning offer a practical bridge between speed and accuracy for field-scale screening. Automated geometric morphometrics and deep learning can be used to locate forewing landmarks and classify subspecies or lineages rapidly and reproducibly, reducing manual effort while leveraging existing morphometric references [3,19]. Public wing-image resources are growing but remain limited, underscoring the need for open, geographically diverse reference sets to improve model generalization [20].

In this study, we developed a classification framework that integrates standardized morphometrics with XGBoost to provide an accessible and interpretable tool for entomologists. We validated this framework by applying it to the challenging case study of identifying the Hunchun bee (*Apis mellifera*) population from morphologically similar subspecies. This work provides a scalable, user-friendly system for insect morphometrics, highlighting the potential of gradient-boosting methods as a supplemental approach for subspecies identification.

## 2. Materials and Methods

### 2.1. The XGBoost-Based Classification Framework

A classification framework was developed using Python 3.10.13 to create custom morphometric classifiers. The framework is designed to accept user-provided data to distinguish a target subspecies from a background population. In this framework, “background population” was defined as the negative class for binary classification. To ensure high model generalizability, this population was constructed as a composite mixture of multiple non-target subspecies, specifically those that are morphologically similar or geographically relevant, rather than a single reference lineage.

The system requires two primary inputs: (1) continuous and categorical variable tables for the target subspecies, and (2) corresponding data for the background subspecies. The target subspecies must be specified by the user. Upon data submission, the framework executes an integrated training procedure. An XGBoost classifier is first trained on the complete feature set. Feature importance scores are then calculated based on the Gain and Cover metrics. The top 10 most contributive features are automatically selected to form a reduced feature set. A final, compact classifier (the “compact model”) is subsequently trained using only these selected features. The output of this process includes the trained model, a visualization of the receiver operating characteristic (ROC) curve, and a list of the top-ranked features. A separate prediction module is provided for classifying new samples automatically, presenting the user with the list of key features identified during training. Prediction is performed by inputting the corresponding morphometric values for a new specimen, upon which the framework returns a subspecies classification.

### 2.2. Case Study: Identification of the Hunchun Honey Bee Population

The dataset used in this study consisted of various morphological features of *Apis mellifera* subspecies maintained at the China National Bee Gene Bank (Jilin City, China; longitude 126.67° E, latitude 43.72° N). The target subspecies was the Hunchun bee, while the background population was composed of a diverse mixture of six other subspecies: *Apis mellifera anatoliaca*, *Apis mellifera carpatica*, *Apis mellifera carnica*, *Apis mellifera caucasica*, the Northeast bee, and the Xinjiang bee. It is important to note that the Hunchun bee, Northeast bee, and Xinjiang bee are officially recognized locally adapted populations within the Chinese National Bee Genetic Resources framework. Although they fall within the broader *Apis mellifera* lineage, they have evolved distinct morphological characteristics due to long-term geographic isolation and acclimatization. In this study, we treated them as distinct classification units because distinguishing these valuable local resources from imported commercial subspecies is a primary objective of our conservation efforts. These subspecies were specifically selected due to their overlapping morphological traits (e.g., dark body color) and geographical relevance, providing a challenging baseline to rigorously validate the XGBoost model’s discrimination capability of the XGBoost model. The raw data were collected by personnel who underwent training prior to data collection, following the guidelines provided in the second volume of the operating manual for the Third National Census of Livestock and Poultry Genetic Resources Survey Form for the Bee Genetic Resources system for collecting the physical trait measurements. To ensure genetic purity and eliminate age-related variability, we implemented a strict controlled sampling protocol. For each subspecies or population, 10 distinct colonies were randomly selected. In each colony, a new empty comb was introduced, and the queen was restricted to laying eggs on this comb for 24 h. After 21 days of development, newly emerged worker bees (1 day old) were collected directly from the comb. To minimize the impact of environmental plasticity and seasonal dimorphism, all specimens were collected during the active beekeeping season (July to August) to exclude winter cohorts. A total of 15 worker bees were sampled from each of the 10 colonies (n = 150 per subspecies). Collected specimens were anesthetized using carbon dioxide (CO_2_) and immediately preserved in 75% ethanol for subsequent dissection and morphometric analysis. The dataset included both continuous and categorical variables. A total of 40 features were collected, encompassing the most comprehensive set of bee morphological traits, including body segment dimensions, appendage measurements, and wing system metrics.

The dataset underwent preprocessing to ensure consistency and readiness for analysis. Missing values were removed, and variable formats were standardized. Continuous variables were normalized using min–max scaling to ensure that each feature contributed equally to the model’s performance. Categorical variables were transformed using one-hot encoding, creating binary columns to represent each category, which facilitated their inclusion in machine learning algorithms. After preprocessing, the dataset was split into a training set (60%) and a validation set (40%).

### 2.3. Feature Selection and Model Training

Classification was performed using XGBoost, a gradient-boosting machine learning model designed for classification tasks in high-dimensional datasets. Initially, a full-feature model was trained on the entire dataset to establish baseline performance. During training, the model used 100 boosting rounds, a learning rate of 0.3, and a maximum tree depth of 15 to balance model complexity and overfitting. The minimum child weight was set to 1 to avoid underfitting, and the subsample was set to 1, meaning the full training set was used to train each tree. Column sampling at each tree split was configured at 80% of the features. Furthermore, to address the class imbalance between the Hunchun bee and background populations (a ratio of approximately 1:7), we explicitly configured the scale_pos_weight parameter, setting it to the ratio of negative to positive instances in the training set to ensure that the model assigned higher penalty weights to misclassifications of the minority class without the need for synthetic resampling. For the baseline models, preliminary validation indicated that the standard hyperparameter configurations yielded optimal performance for this dataset; therefore, these validated settings were utilized. Specifically, the Random Forest model was configured with ntree = 500 and the default variable sampling rate (mtry). The Support Vector Machine (SVM) employed a radial basis function (RBF) kernel with default cost (C = 1) and gamma parameters.

Feature importance was calculated using the built-in ranking mechanism of XGBoost, which ranks features based on their Gain and Cover metrics. Gain refers to the improvement in model performance when a feature is used in a split, while Cover measures the relative number of samples affected by each feature. The top 10 most contributive features were selected based on these importance scores.

A secondary compact model, referred to as the compact model, was then trained using only the top 10 features. The reduced dataset was re-split into a training set and validation set with the same 60/40 ratio, and the XGBoost model was retrained using the reduced feature set. Both the full-feature model and the compact model were independently evaluated on their respective validation sets.

### 2.4. Dimensionality Reduction

To assess the separability of Hunchun bees from the other subspecies, Factor Analysis of Mixed Data (FAMD) was applied for dimensionality reduction. This method combines both continuous and categorical features into a set of principal components, which allows for visualization in a lower-dimensional space.

### 2.5. Model Evaluation and Cross-Validation

The model’s performance was evaluated using multiple metrics—accuracy, precision, recall, F1-score, and AUC–receiver operating characteristic (ROC)—in terms of its ability to classify Hunchun bees and other subspecies accurately. To rigorously evaluate the generalization capability and stability of our classification framework, we employed a five-fold cross-validation scheme. This procedure involved dividing the data into five subsets, utilizing each subset once as a validation set while the remaining four served as the training set. To assess the robustness of the feature selection strategy, the feature ranking and selection process was performed independently within the training fold of each iteration. This procedure ensures that the calculated evaluation metrics reflect the model’s true ability to identify discriminatory features on unseen data, strictly preventing selection bias and data leakage. This process was repeated five times, with each subset being used for testing once.

### 2.6. Statistical Analysis

The significance of differences in continuous variables across subspecies was assessed using Analysis of Variance (ANOVA) to test for statistical differences in feature means. The *p*-values for various features were computed to determine the statistical significance of the differences. For categorical variables, Chi-square tests were used to assess whether the distribution of categories differed significantly across subspecies.

The machine learning models were implemented using the R programming language (version 4.4.1). Statistical tests and model evaluations were conducted within this environment. All computations were performed on a local machine with a standard configuration. The analysis code is available for review on figshare (10.6084/m9.figshare.30520946).

## 3. Results

### 3.1. Overall Classification Workflow

**Figure 1:**
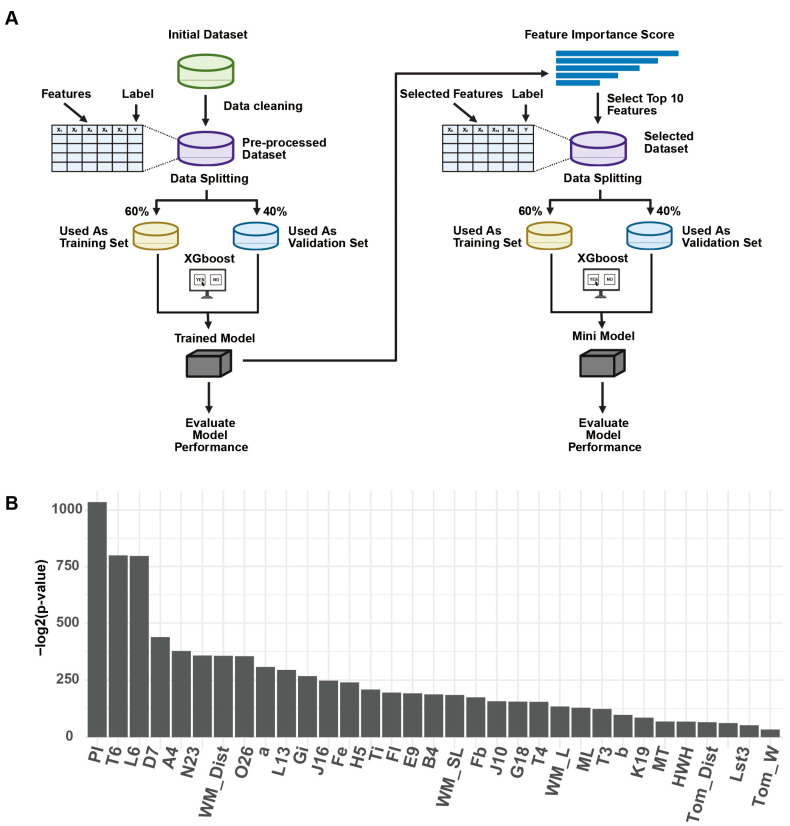
Overall classification workflow and results of ANOVA for different bee subgroups. (**A**) End-to-end pipeline: standardized morphometric acquisition (40 traits), preprocessing, embedded feature selection with XGBoost, model training, and evaluation. (**B**) One-way ANOVA across subspecies for continuous traits, ranked by −log2(p); Abbreviations: Pl: Proboscis length; T6: Width of sternite 6; L6: Length of sternite 6; D7, A4, N23, L13, O26, J16, E9, B4, J10, G18, K19: Forewing vein angles; WM_Dist: Distance between wax mirrors on sternite 3; a, b: Cubital vein a and b; Gi: Cubital index; Fe: Length of hind leg femur; H5: Length of hair cover (tergite 5); Ti: Length of hind leg tibia; Fl: Length of forewing; WM_SL: Slant length of wax mirror on sternite 3; Fb: Width of forewing; T4: Length of tergite 4; WM_L: Length of wax mirror on sternite 3; ML: Length of hind leg metatarsus; T3: Length of tergite 3; MT: Width of hind leg metatarsus; HWH: Number of hindwing hooks; Tom_Dist: Distance from tomentum to posterior margin of tergite 4; Lst3: Length of sternite 3; Tom_W: Width of tomentum (tergite 4).

Figure 1A illustrates the overall workflow of the proposed classification framework. The process begins with the importing of raw datasets, followed by standardized data cleaning and preprocessing to remove missing values and unify variable formats. The preprocessed dataset is subsequently split into a training set (60%) and a validation set (40%). An initial XGBoost classifier is trained on the full feature set to assess baseline classification performance.

Feature importance scores are then calculated based on the trained model, and the top 10 most contributive features are extracted. A secondary compact model (compact model) is constructed using only these selected features. The reduced dataset is re-split into training and validation sets (60/40), and XGBoost is retrained to build the compact model. Both models are independently evaluated on their respective validation sets. This framework integrates automated hyperparameter tuning and built-in visualization of feature importance scores, thereby combining model optimization and interpretability within a unified pipeline. The architecture of both models is identical, with the only difference being the dimensionality of the input features.

### 3.2. Case Study Data Characteristics

In the global comparison of continuous traits, one-way ANOVA across subspecies revealed a wide spread of significance when expressed as −log2(p), with proboscis length showing the strongest signal and the distance “from tomentum to posterior margin (tergite 4)” showing the weakest (Figure 1B).

**Figure 2:**
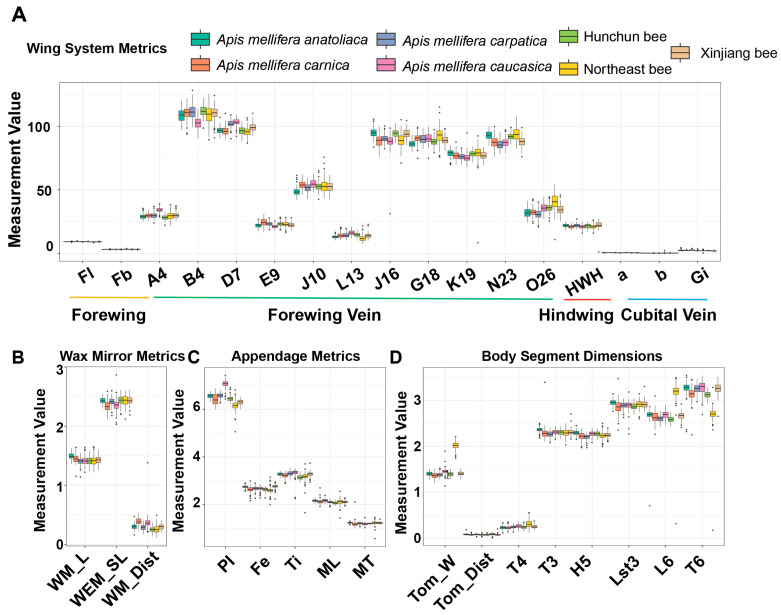
Distribution characteristics of continuous variable data. (**A**) The distribution of wing system metrics among different bee subgroups. (**B**) The distribution of wax mirror metrics among different bee subgroups. (**C**) The distribution of appendage metrics among different bee subgroups. (**D**) The distribution of body segment dimensions among different bee subgroups. Abbreviations: (**A**) Fl: Length of forewing; Fb: Width of forewing; Gi: Cubital index; HWH: Number of hindwing hooks; a: Cubital vein a; b: Cubital vein b; A4–O26: Forewing vein angles. (**B**) WM_L: Length of wax mirror on sternite 3; WEM_SL: Slant length of wax mirror on sternite 3; WM_Dist: Distance between wax mirrors on sternite 3. (**C**) Pl: Proboscis length; Fe: Length of hind leg femur; Ti: Length of hind leg tibia; ML: Length of hind leg metatarsus; MT: Width of hind leg metatarsus. (**D**) Tom_W: Width of tomentum (tergite 4); Tom_Dist: Distance from tomentum to posterior margin of tergite 4; T4: Length of tergite 4; T3: Length of tergite 3; H5: Length of hair cover (tergite 5); Lst3: Length of sternite 3; L6: Length of sternite 6; T6: Width of sternite 6.

Wing system metrics displayed visible between-subspecies differences but retained substantial overlap, indicating that no single wing-based variable can serve as a standalone discriminator (Figure 2A). Wax mirror measurements varied across subspecies yet showed broad interquartile overlap, suggesting limited individual utility (Figure 2B). Within the appendage group, proboscis length showed the most clear separation—consistent with its significance being the highest in ANOVA—whereas several hind-leg measures exhibited comparatively modest shifts (Figure 2C). Body segment dimensions showed moderate separation overall; the “from tomentum to posterior margin (tergite 4)” measure contributed the least, aligning with its lowest significance in the omnibus screen (Figure 2D).

**Figure 3:**
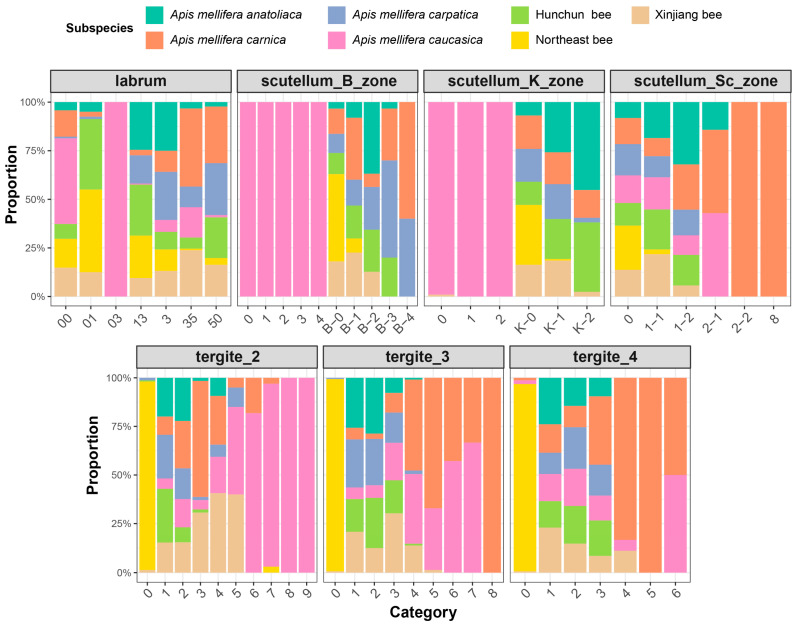
Distribution characteristics of categorical variable data.

For categorical traits, subspecies compositions differed but remained strongly overlapping for the labrum categories, and no single category was unique to one subspecies. A similar pattern was observed in the scutellum_B_zone, where proportion differences were present yet not decisive for discrimination. The scutellum_K_zone showed shifts in category proportions across subspecies, but categories were broadly shared and did not yield subspecies-specific signatures. The scutellum_Sc_zone displayed the same overlapping structure, indicating that single categories were insufficient to reliably separate Hunchun bees from the other subspecies (Figure 3).

### 3.3. Case Study Model Performance

**Figure 4:**
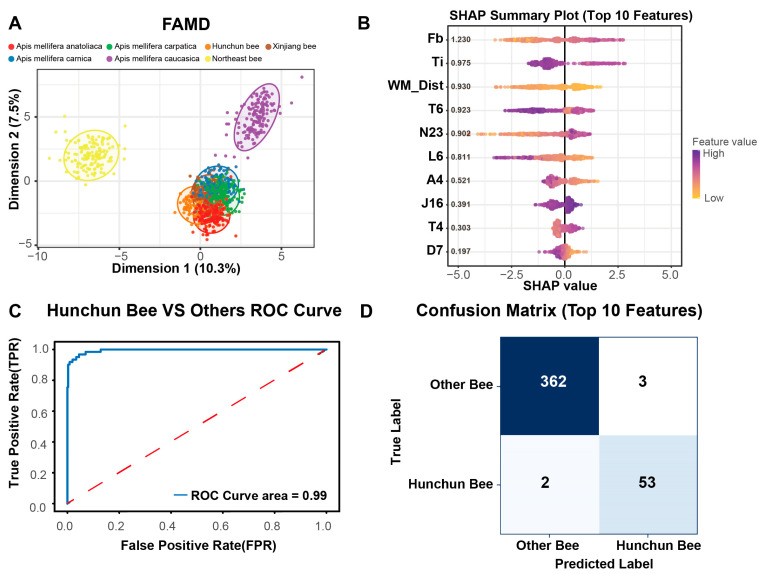
Classification prediction results. (**A**) FAMD ordination (first two components) shows overlap between Hunchun and other black-colored subspecies. (**B**) SHAP summary plot (Beeswarm) for the top 10 features. The features are ordered by their global importance (mean absolute SHAP value). Each dot represents a single sample. The color indicates the feature value (Purple = High, Yellow = Low). The *x*-axis (SHAP value) shows the impact of the feature on the model’s output: positive values indicate a higher likelihood of being classified as a Hunchun bee, while negative values indicate a higher likelihood of being classified as the background population. (**C**) ROC curve for the compact model (10 features) with AUC, and the red dashed line represents the performance of a random classifier. (**D**) Confusion matrix for the initial validation set (40% hold-out split).

Factor Analysis of Mixed Data (FAMD) was used for dimensionality reduction analysis, with Hunchun bees showing substantial overlap with other black-colored honey bee subspecies in the first two principal components (Figure 4A); the data distribution of the former could not be linearly separated from that of *Apis mellifera caucasica* or *Apis mellifera carnica*. To improve classification performance, embedded feature selection was applied using XGBoost gain scores. For the compact model, we performed feature importance analysis on the entire training dataset to determine the top 10 features embedded in the final tool. It is noteworthy that stability analysis during our cross-validation procedure confirmed high consistency: these specific features were repeatedly selected as top contributors across independent cross-validation folds, validating their biological relevance and the robustness of the selection method. The SHAP summary plot interprets the impact of the top 10 selected features on the model’s predictions (Figure 4B). The width of the forewing (Fb) was identified as the most influential feature; higher values of this trait correspond to positive SHAP values, significantly increasing the probability of identifying a specimen as a Hunchun bee. Conversely, features such as the width of sternite 6 showed a different pattern, where higher values tended to push the prediction towards the background population (negative SHAP values). Using the top 10 features, the classifier achieved an AUC of 0.99 (Figure 4C), indicating high discrimination in separating Hunchun bees from other subspecies. Confusion matrix analysis on the static validation set (40% hold-out) showed that the model correctly identified 53 of 55 Hunchun bee samples and 362 of 365 other bee samples (Figure 4D). A post hoc inspection revealed that these misclassifications were exclusively confined to the Hunchun bee and two specific background subspecies: *Apis mellifera carnica* and *Apis mellifera caucasica*. These specimens represent ‘edge cases’—individuals whose specific trait values deviated from their population means, placing them in the boundary region of the morphospace shared by these closely related lineages. Crucially, comprehensive performance evaluation using 5-fold cross-validation yielded a higher average Recall of 0.994 (Table 1), indicating that the misclassifications observed in the hold-out set represent the lower bound of the model’s performance variability, largely driven by specific sampling distributions in that single split.

### 3.4. XGBoost Outperforms Other Models in Cross-Validation

To further assess model generalizability, five-fold cross-validation was performed across multiple classification algorithms: XGBoost, Support Vector Machine (SVM), and Random Forest. Key evaluation metrics—accuracy, precision, recall, F1-score, and AUC—were computed along with their 95% confidence intervals (Table 1). XGBoost achieved the highest accuracy (0.982; 95% CI: 0.972–0.992) and F1-score (0.990; 95% CI: 0.984–0.995), as well as high recall (0.994) and precision (0.985), resulting in an AUC of 0.997 (95% CI: 0.995–0.999). Random Forest yielded comparable recall (1.000; 95% CI: 1.000–1.000) and AUC (0.996), but slightly lower precision and accuracy. SVM performed reasonably well but showed the lowest accuracy (0.946) and F1-score (0.969) among the three.

Collectively, while all three classifiers achieved relatively high performance, XGBoost consistently outperformed baseline models across all major evaluation metrics under cross-validation, demonstrating its robustness and suitability for morphological classification of Hunchun bee.

## 4. Discussion

This study presents a standardized morphometric workflow for assigning *Apis mellifera* subspecies using a compact set of routinely measured traits, following community guidance on sampling and measurement consistency [2,21]. The workflow is designed as a screening tool that complements classical taxonomy and genetic assays, not as a replacement [2,21,22,23]. In practice, rapid morphometric triage can guide field surveys and breeding choices, with genetic tests reserved for uncertain or management-critical cases [22,23,24]. Specifically, we recommend prioritizing this morphometric framework in cases of large-scale field monitoring and preliminary breeding screening, where processing speed and cost-efficiency are critical. Conversely, molecular verification remains essential for high-stakes management decisions, such as certifying purebred conservation stocks or resolving samples with borderline probability scores that suggest potential admixture.

The compact model prioritized forewing width and several venation angles (A4, N23, D7, J16), together with abdominal plate measures (S6, T4), the distance between wax mirrors on sternite 3, and hind-leg tibia length. These features come from the classical Ruttner character set and its later standardizations [2,21]. Forewing venation and shape capture stable, population-level structure and often align with broad genetic patterns [22,25,26]. The geometric morphometrics of wings can discriminate between subspecies and mirror population structures seen with microsatellites or SNPs [22,25,26]. Abdominal metrics extend information beyond wings within standard Ruttner/COLOSS panels and are widely used in regional surveys and keys [27,28]. Body-size traits such as tibia length show appreciable heritability in workers, which supports their repeated contribution to discriminant functions [29]. Surveys across Africa and the Mediterranean, and in hybrid or contact zones, show that venation-based shape spaces separate regional subspecies while also revealing clinal or admixed structures [25,26,30,31]. Together, these prioritized features show complementary signals—wing geometry, thoraco-abdominal proportions, and leg segment length—yielding interpretable assignments without invoking untested functional claims.

Gradient-boosted trees fit tabular morphometric data because they show strong performance when fitting tabular morphometric data, modeling non-linearities and feature interactions with minimal preprocessing and perform strongly on such data [32,33]. Compared with linear models, XGBoost adds L1/L2 regularization, learning-rate shrinkage, and row/column subsampling, which help control complexity and curb overfitting while retaining predictive accuracy [33]. Unlike SVMs, which often require full data or explicit imputation, XGBoost uses sparsity-aware splitting with a learned default path for missing values, reducing preprocessing and preserving information. Model-side importance can be complemented with SHAP values for global and case-level explanations, allowing predictions to be traced back to specific traits [34]. To address class imbalance, common in biodiversity datasets, XGBoost supports class weighting (e.g., scale_pos_weight) and AUC-based evaluation, providing straightforward controls without resampling. In this study, these properties motivated our choice of XGBoost as the primary classifier, while leaving room for confirmatory analyses with alternative algorithms where needed.

Because the feature dictionary is decoupled from the trained booster, the implementation can be ported to other taxa with standardized linear or landmark schemes, contingent on representative training data and harmonized protocols. Candidate systems include Bombus and other Hymenoptera, and taxa in Coleoptera and Lepidoptera where routine morphometric pipelines exist. Cross-site validation and, where appropriate, domain-adaptation strategies can help manage distributional shift during deployment.

Nevertheless, this study possesses certain limitations, mainly related to the characteristics of our test dataset. First, the sampling covered a limited set of regions and habitats. This may increase sensitivity to local morphometric patterns and restrict generalizability. Second, environmental and developmental plasticity—temperature, nutrition, season, and worker age—can change wing venation angles and body metrics. This may introduce batch-to-batch drift. Third, manual landmarking and measurements can vary across operators, adding random error and lowering the performance ceiling. These constraints pertain to the dataset used here rather than the approach itself. Future iterations could mitigate them by establishing larger-scale, multi-regional datasets that capture broader biological variation. To address the limitation of manual measurement error, we recommend integrating automated geometric morphometrics or deep learning-based measurement tools into the workflow [3,19]. These technologies can standardize data acquisition and significantly increase throughput. By combining such high-quality, high-volume data with our XGBoost framework, we anticipate that the model’s performance could approach the theoretical biological limit of subspecies differentiation, minimizing the trade-off between speed and precision.

## 5. Conclusions

In this study, we developed and validated an XGBoost-based morphometric classification framework for the automated identification of western honey bee (*Apis mellifera*) subspecies. Employing a compact set of 10 conventionally measured morphometric features, our “compact model” achieved high classification performance (Accuracy = 0.98, AUC = 0.99) and outperformed baseline models, including Support Vector Machine and Random Forest. This research provides not only a static model but, more significantly, a scalable and interpretable workflow. Researchers can utilize this framework, supported by a user-friendly software interface, to create customized classifiers by integrating their own morphometric data, extending its application to other *Apis mellifera* subspecies or different insect taxa. This XGBoost-based morphometric classification system offers an efficient and deployable tool to support bee biology research, breeding practices, and conservation strategies. By facilitating subspecies identification, this system contributes to efforts to protect the genetic integrity of *Apis mellifera* and promote its sustainable management on a global scale.

## Figures and Tables

**Table 1:** Comparison of Prediction Accuracy of Different Machine Learning Models.

Model	XGBoost	SVM	Random Forest
Accuracy	0.982 (0.972, 0.992)	0.946 (0.903, 0.989)	0.962 (0.943, 0.981)
Precision	0.985 (0.974, 0.996)	0.956 (0.921, 0.991)	0.958 (0.937, 0.978)
Recall	0.994 (0.985, 1.004)	0.982 (0.968, 0.996)	1.000 (1.000, 1.000)
F1	0.990 (0.984, 0.995)	0.969 (0.944, 0.993)	0.978 (0.968, 0.989)
AUC	0.997 (0.995, 0.999)	0.957 (0.909, 1.005)	0.996 (0.993, 0.999)

## Data Availability

The original data presented in the study are openly available in FigShare at DOI 10.6084/m9.figshare.30520946.

## Abbreviations

The following abbreviations are used in this manuscript:

a	Cubital vein a
A4	Forewing vein angle A4
ANOVA	Analysis of Variance
AUC	Area Under the (ROC) Curve
b	Cubital vein b
B4	Forewing vein angle B4
CI	Confidence Interval
CV	Cross-Validation
D7	Forewing vein angle D7
DCA	Drone Congregation Area(s)
DWM	Distance between wax mirrors on sternite 3
E9	Forewing vein angle E9
F1	F1-score
FAMD	Factor Analysis of Mixed Data
Fb	Width of forewing
FDR	False Discovery Rate
Fe	Length of hind leg femur
Fl	Length of forewing
FPR	False Positive Rate
G18	Forewing vein angle G18
Gi	Cubital index (a/b)
Gi	Cubital index ($Ci = a/b$)
GIS/GPS	Geographic Information System/Global Positioning System
H5	Length of hair cover on tergite 5
HWH	Number of hindwing hooks (Hamuli)
IQR	Interquartile Range
J10	Forewing vein angle J10
J16	Forewing vein angle J16
K19	Forewing vein angle K19
L13	Forewing vein angle L13
L3	Length of sternite 3
L6	Length of sternite 6
Lst3	Length of sternite 3
LWM	Length of wax mirror on sternite 3
ML	Length of hind leg metatarsus
MT	Width of hind leg metatarsus
N23	Forewing vein angle N23
O26	Forewing vein angle O26
PCA	Principal Component Analysis
Pl	Proboscis length
QC	Quality Control
RF	Random Forest
ROC	Receiver Operating Characteristic
SD	Standard Deviation
SE	Standard Error
SHAP	SHapley Additive exPlanations
Slant LWM	Slant length of wax mirror on sternite 3
SNP	Single-Nucleotide Polymorphism
SOP	Standard Operating Procedure
SVM	Support Vector Machine
T3	Length of tergite 3
T4	Length of tergite 4
T6	Width of sternite 6
Ti	Length of hind leg tibia
Tom_Dist	Distance from tomentum to posterior margin
Tom_W	Width of tomentum on tergite 4
TPR	True Positive Rate
WM_Dist	Distance between wax mirrors on sternite 3
WM_L	Length of wax mirror on sternite 3
WM_SL	Slant length of wax mirror on sternite 3
XGBoost	Extreme Gradient Boosting

## References

[1] Hung K.-L.J., Kingston J.M., Albrecht M., Holway D.A., Kohn J.R. (2018). The Worldwide Importance of Honey Bees as Pollinators in Natural Habitats. Proc. Biol. Sci..

[2] Meixner M.D., Pinto M.A., Bouga M., Kryger P., Ivanova E., Fuchs S. (2013). Standard Methods for Characterising Subspecies and Ecotypes of *Apis mellifera*. J. Apic. Res. Bee World.

[3] Nawrocka A., Kandemir İ., Fuchs S., Tofilski A. (2018). Computer Software for Identification of Honey Bee Subspecies and Evolutionary Lineages. Apidologie.

[4] Ángel Beamonte E., Martín Ramos P., Santolaria P., Sales E., Abizanda J., Yániz J.L. (2018). Automatic Determination of Landmark Coordinates for Honey Bee Forewing Venation Using a New MATLAB-Based Tool. J. Apic. Res..

[5] Amiri E., Abou-Shaara H., McAfee A. (2024). The Effect of Major Abiotic Stressors on Honey Bee (*Apis mellifera* L.) Queens and Potential Impact on Their Progeny. Apidologie.

[6] Galindo-Cardona A., Carolina Monmany A., Moreno-Jackson R., Rivera-Rivera C., Huertas-Dones C., Caicedo-Quiroga L., Giray T. (2012). Landscape Analysis of Drone Congregation Areas of the Honey Bee, *Apis mellifera*. J. Insect Sci..

[7] Ayup M.M., Gärtner P., Agosto-Rivera J.L., Marendy P., de Souza P., Galindo-Cardona A. (2021). Analysis of Honeybee Drone Activity during the Mating Season in Northwestern Argentina. Insects.

[8] Steed E.J., Painting C.J., Mortensen A.N. (2025). Global Variation in Honey Bee (*Apis mellifera*) Mating Flight Times. N. Z. J. Zool..

[9] Muñoz I., Henriques D., Johnston J.S., Chávez-Galarza J., Kryger P., Pinto M.A. (2015). Reduced SNP Panels for Genetic Identification and Introgression Analysis in the Dark Honey Bee (*Apis mellifera mellifera*). PLoS ONE.

[10] Henriques D., Parejo M., Vignal A., Wragg D., Wallberg A., Webster M.T., Pinto M.A. (2018). Developing Reduced SNP Assays from Whole-Genome Sequence Data to Estimate Introgression in an Organism with Complex Genetic Patterns, the Iberian Honeybee (*Apis mellifera iberiensis*). Evol. Appl..

[11] Qiu L., Dong J., Li X., Parey S.H., Tan K., Orr M., Majeed A., Zhang X., Luo S., Zhou X. (2023). Defining Honeybee Subspecies in an Evolutionary Context Warrants Strategized Conservation. Zool. Res..

[12] Momeni J., Parejo M., Nielsen R.O., Langa J., Montes I., Papoutsis L., Farajzadeh L., Bendixen C., Căuia E., Charrière J.-D. (2021). Authoritative Subspecies Diagnosis Tool for European Honey Bees Based on Ancestry Informative SNPs. BMC Genom..

[13] Donthu R., Marcelino J.A.P., Giordano R., Tao Y., Weber E., Avalos A., Band M., Akraiko T., Chen S.-C., Reyes M.P. (2024). HBeeID: A Molecular Tool That Identifies Honey Bee Subspecies from Different Geographic Populations. BMC Bioinform..

[14] Muñoz I., Henriques D., Jara L., Johnston J.S., Chávez-Galarza J., De La Rúa P., Pinto M.A. (2017). SNPs Selected by Information Content Outperform Randomly Selected Microsatellite Loci for Delineating Genetic Identification and Introgression in the Endangered Dark European Honeybee (*Apis mellifera mellifera*). Mol. Ecol. Resour..

[15] Henriques D., Lopes A.R., Chejanovsky N., Dalmon A., Higes M., Jabal-Uriel C., Le Conte Y., Reyes-Carreño M., Soroker V., Martín-Hernández R. (2021). A SNP Assay for Assessing Diversity in Immune Genes in the Honey Bee (*Apis mellifera* L.). Sci. Rep..

[16] Techer M.A., Clémencet J., Simiand C., Preeaduth S., Azali H.A., Reynaud B., Hélène D. (2017). Large-Scale Mitochondrial DNA Analysis of Native Honey Bee *Apis mellifera* Populations Reveals a New African Subgroup Private to the South West Indian Ocean Islands. BMC Genet..

[17] Utzeri V.J., Ribani A., Taurisano V., Banqué C.H.i., Fontanesi L. (2021). Distribution of the Main *Apis mellifera* Mitochondrial DNA Lineages in Italy Assessed Using an Environmental DNA Approach. Insects.

[18] Oleksa A., Kusza S., Tofilski A. (2021). Mitochondrial DNA Suggests the Introduction of Honeybees of African Ancestry to East-Central Europe. Insects.

[19] García C.A.Y., Rodrigues P.J., Tofilski A., Elen D., McCormak G.P., Oleksa A., Henriques D., Ilyasov R., Kartashev A., Bargain C. (2022). Using the Software DeepWings© to Classify Honey Bees across Europe through Wing Geometric Morphometrics. Insects.

[20] Oleksa A., Căuia E., Siceanu A., Puškadija Z., Kovačić M., Pinto M.A., Rodrigues P.J., Hatjina F., Charistos L., Bouga M. (2023). Honey Bee (*Apis mellifera*) Wing Images: A Tool for Identification and Conservation. Gigascience.

[21] Ruttner F. (1988). Biogeography and Taxonomy of Honeybees.

[22] Miguel I., Baylac M., Iriondo M., Manzano C., Garnery L., Estonba A. (2011). Both Geometric Morphometric and Microsatellite Data Consistently Support the Differentiation of the *Apis mellifera* M Evolutionary Branch. Apidologie.

[23] Nielsen D.I., Ebert P.R., Hunt G.J., Guzmán-Novoa E., Kinnee S.A., Page R.E. (1999). Identification of Africanized Honey Bees (Hymenoptera: Apidae) Incorporating Morphometrics and an Improved Polymerase Chain Reaction Mitotyping Procedure. Ann. Entomol. Soc. Am..

[24] Rinderer T.E., Buco S.M., Rubink W.L., Daly H.V., Stelzer J.A., Riggio R.M., Baptista F.C. (1993). Morphometric Identification of Africanized and European Honey Bees Using Large Reference Populations. Apidologie.

[25] Henriques D., Chávez-Galarza J., S. G. Teixeira J., Ferreira H., J. Neves C., Francoy T.M., Pinto M.A. (2020). Wing Geometric Morphometrics of Workers and Drones and Single Nucleotide Polymorphisms Provide Similar Genetic Structure in the Iberian Honey Bee (*Apis mellifera iberiensis*). Insects.

[26] Francoy T.M., Wittmann D., Drauschke M., Müller S., Steinhage V., Bezerra-Laure M.A.F., De Jong D., Gonçalves L.S. (2008). Identification of Africanized Honey Bees through Wing Morphometrics: Two Fast and Efficient Procedures. Apidologie.

[27] Crewe R.M., Hepburn H.R., Moritz R.F.A. (1994). Morphometric Analysis of 2 Southern African Races of Honeybee. Apidologie.

[28] Kuliçi M., Shehu L. (2021). Estimation of 14 Morphological Traits of Honey Bees in Tropoja District and Their Correlation with Honey Production. J. Hyg. Eng. Des..

[29] Oldroyd B., Rinderer T., Buco S. (1991). Heritability of Morphological Characters Used to Distinguish European and Africanized Honeybees. Theor. Appl. Genet..

[30] Nazzi F. (1992). Morphometric Analysis of Honey Bees from an Area of Racial Hybridization in Northeastern Italy. Apidologie.

[31] Dall’Olio R., Marino A., Lodesani M., Moritz R.F.A. (2007). Genetic Characterization of Italian Honeybees, *Apis mellifera ligustica*, Based on Microsatellite DNA Polymorphisms. Apidologie.

[32] Shwartz-Ziv R., Armon A. (2022). Tabular Data: Deep Learning Is Not All You Need. Inf. Fusion.

[33] Chen T., Guestrin C. XGBoost: A Scalable Tree Boosting System. Proceedings of the 22nd ACM SIGKDD International Conference on Knowledge Discovery and Data Mining.

[34] Lundberg S., Lee S.-I. A Unified Approach to Interpreting Model Predictions. Proceedings of the 31st International Conference on Neural Information Processing Systems.

